# Supplementary figures and images for: Deep learning for automatic head and neck lymph node level delineation provides expert-level accuracy
Source: Front Oncol. 2023 Feb 16;13:1115258. doi: 10.3389/fonc.2023.1115258 (PMC9978473; doi:10.3389/fonc.2023.1115258)

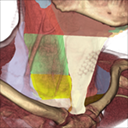

Supplement: Supplementary file 2 [file DataSheet_2.zip › icon.png]
